# Supplementary material for: Crystal and electronic facet analysis of ultrafine Ni2P particles by solid-state NMR nanocrystallography
Source: Nat Commun. 2021 Jul 15;12:4334. doi: 10.1038/s41467-021-24589-5 (PMC8282690; doi:10.1038/s41467-021-24589-5)
Supplement: Supplementary file 1 — Supplementary Information [file 41467_2021_24589_MOESM1_ESM.pdf]

Crystal and electronic facet analysis of ultrafine Ni_2P particles by solid-state NMR nanocrystallography

Wassilios Papawassiliou[1], José P. Carvalho[1], Nikolaos Panopoulos[2], Yasser Al Wahedi[3]*, Vijay Kumar Shankarayya Wadi[3], Xinnan Lu[3], Kyriaki Polychronopoulou[4,5], Jin Bae Lee [6], Sanggil Lee [6], Chang Yeon Kim[6], Hae Jin Kim[6], Marios Katsiotis[3], Vasileios Tzitzios[2], Marina Karagianni[2], Michael Fardis[2], Georgios Papavassiliou[2]* and Andrew J. Pell[1,7]*.

[1] Department of Materials and Environmental Chemistry, Arrhenius Laboratory, Stockholm University, Svante Arrhenius väg 16 C, SE-106 91 Stockholm, Sweden,

[2] Institute of Nanoscience and Nanotechnology, National Center for Scientific Research "Demokritos", 153 10 Aghia Paraskevi, Attiki, Greece,

[3] Department of Chemical Engineering, Khalifa University, PO Box 2533, Abu Dhabi, United Arab Emirates,

[4] Center for Catalysis and Separations (CeCaS), Khalifa University, PO Box 127788, Abu Dhabi, United Arab Emirates,

[5] Department of Mechanical Engineering, Khalifa University, PO Box 127788, Abu Dhabi, United Arab Emirates,

[6] Electron Microscopy Research Center, Korea Basic Science Institute, 169-148 Gwahak-ro, Yuseong-gu, Daejeon 34133, Republic of Korea,

[7] Centre de RMN à Très Hauts Champs de Lyon (UMR 5280 CNRS / ENS Lyon / Université Claude Bernard Lyon 1), Université de Lyon, 5 rue de la Doua, 69100 Villeurbanne, France.

yasser.alwahedi@ku.ac.ae, g.papavassiliou@inn.demokritos.gr, *andrew.pell@mmk.su.se

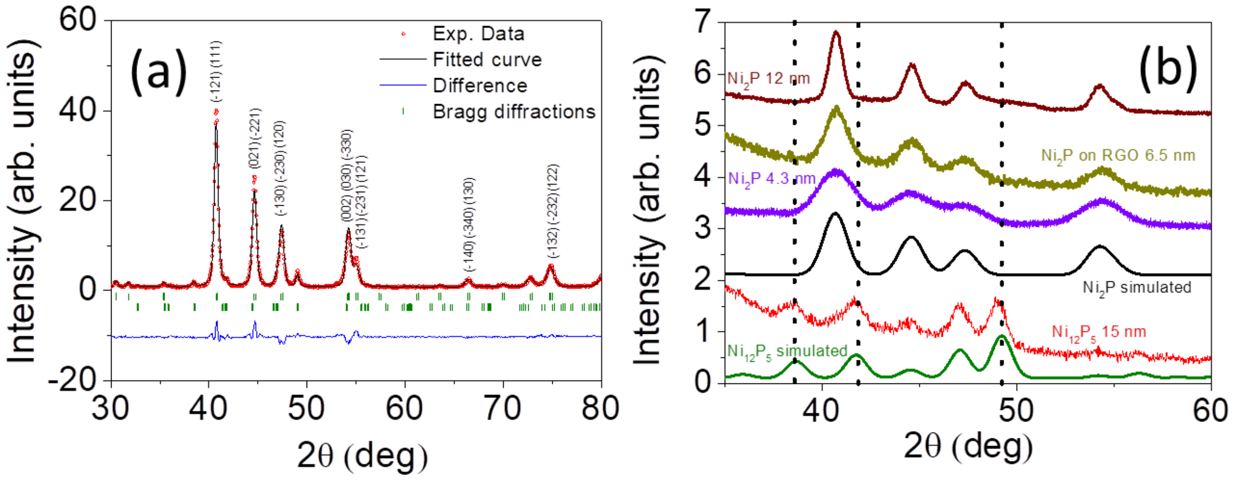

Supplementary Figure 1: (a) The experimental X–ray powder diffraction patterns (red) and the Rietveld analysis (black) of microcrystalline Ni_2P. (b) Comparison of the experimental XRD patterns of the three nanosized Ni_2P systems discussed in the main article (12 nm in brown color, 6.5 nm grown on RGO in olive color, and 4.3 nm in violet color), with the XRD pattern of nanosized $Ni_{12}P_5$ (15 nm in red color). The black (Ni_2P) and green ($Ni_{12}P_5$) XRD patterns are simulations acquired from literature cif files that have been convoluted with a Gaussian to simulate XRDs of nanoparticles. Definitely, no $Ni_{12}P_5$ is present in all three Ni_2P nanoparticle systems.

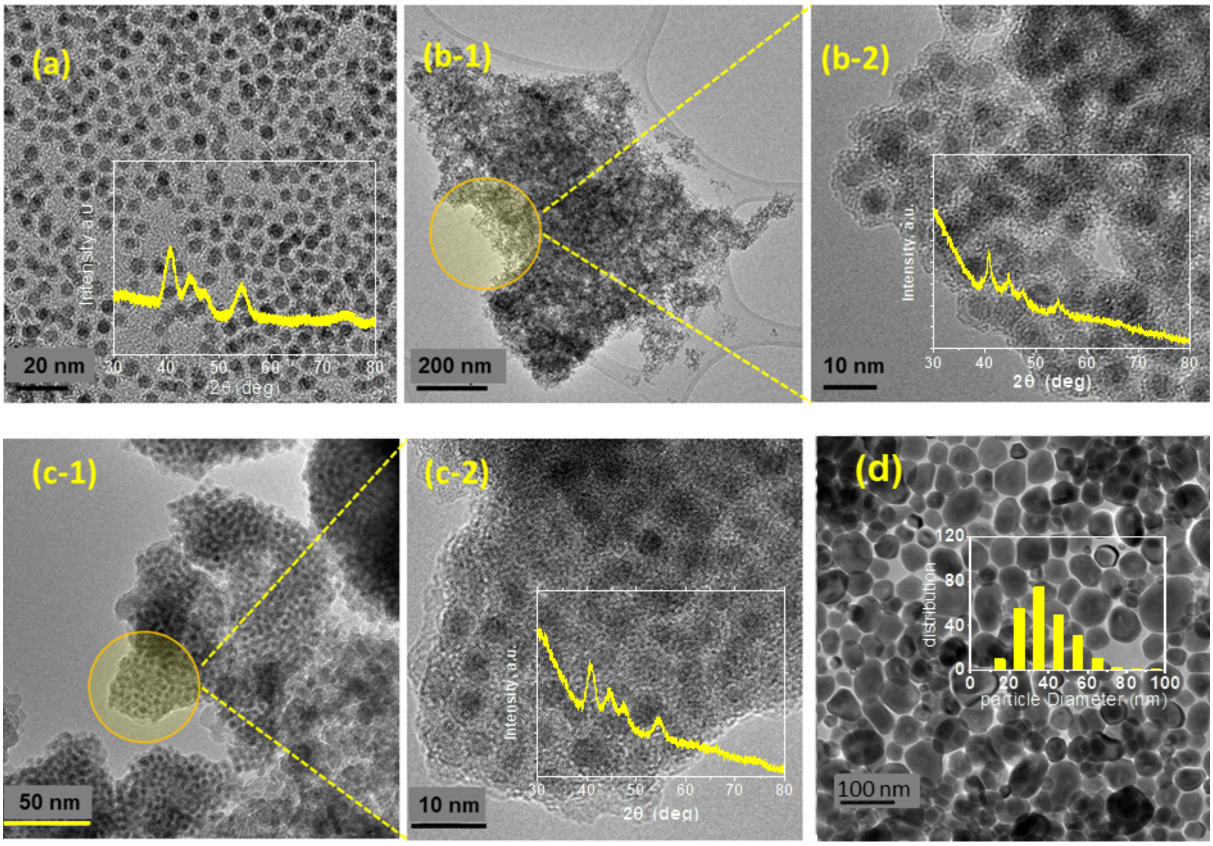

Supplementary Figure 2: **Fabrication of uniform ultra-small Ni_2P nanoparticles:** (a-c) TEM images of three Ni_2P nanoparticle samples with mean size ≈ 4-6 nm. Insets are the relevant XRD patterns. (d) In addition, Ni_2P nanoparticles with mean size ≈ 40 nm were synthesized and ^{31}P MAS NMR results are presented in Figure 2a of the main article.

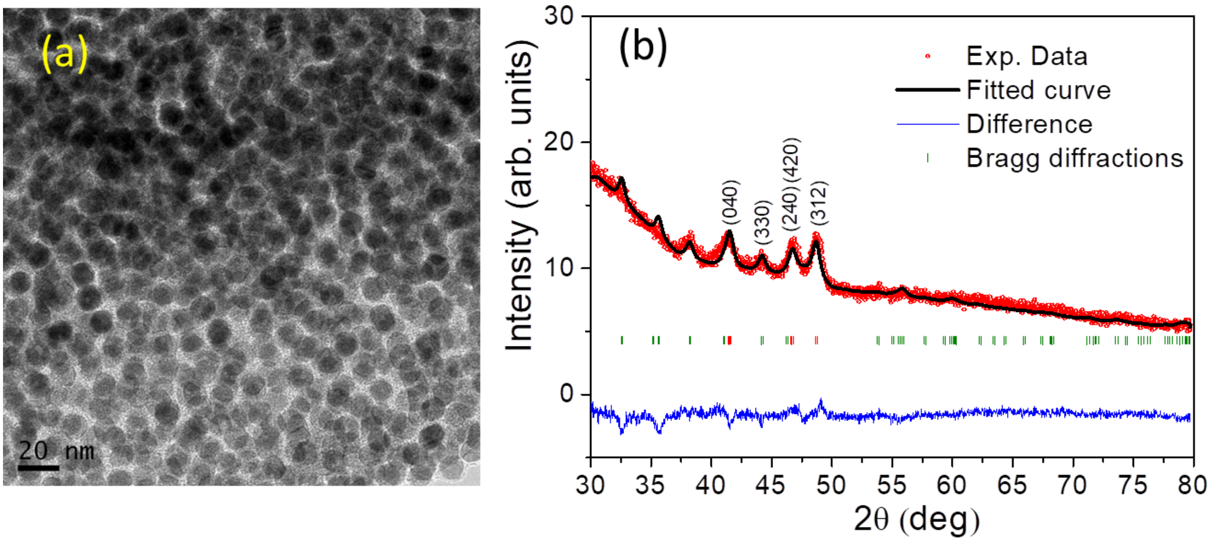

Supplementary Figure 3. **Morphological and structural characterization of $Ni_{12}P_5$ nanoparticles** (a) TEM of the $Ni_{12}P_5$ nanoparticles with average size 15 nm. (b) XRD pattern (red) and Rietveld analysis (black) of the synthesized $Ni_{12}P_5$ nanoparticles.

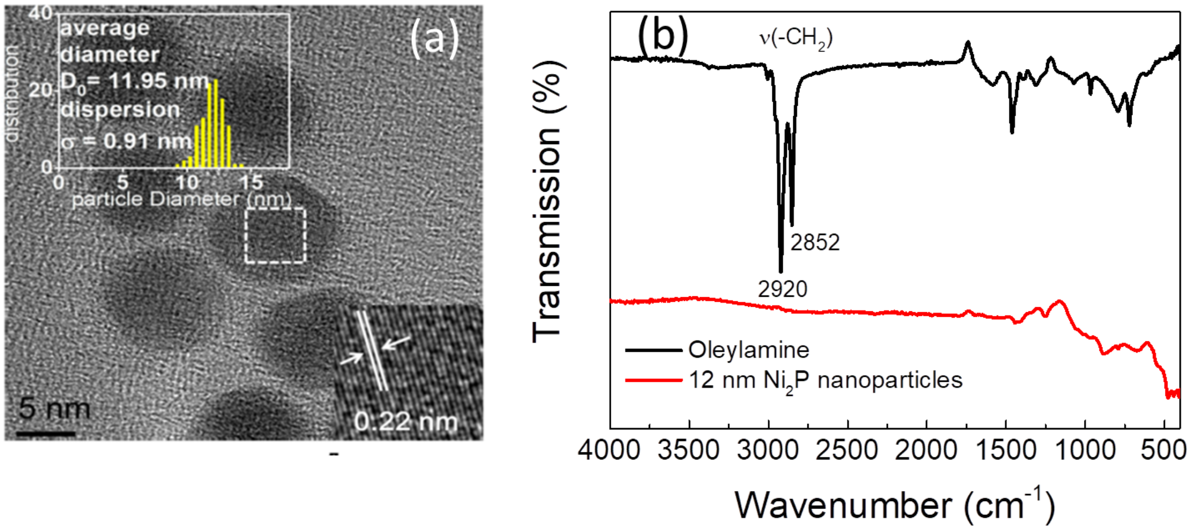

Supplementary Figure 4. TEM and FT-IR of the 12 nm nanocrystalline Ni_2P. (a) TEM and particle size distribution analysis. The measured distance between the lattice fringes corresponds to 2.21Å, which matches with the 111 plane in Ni_2P[35]. (b) FT-IR of the 12 nm Ni_2P in comparison to oleylamine. No traces of oleylamine and trioctylphosphine (TOP) are observed (strong peaks at around 3000 cm^{-1} are assigned to the C-H stretching modes of the CH_2 and CH_3 groups, from oleylamine (trioctylphosphine as well)).

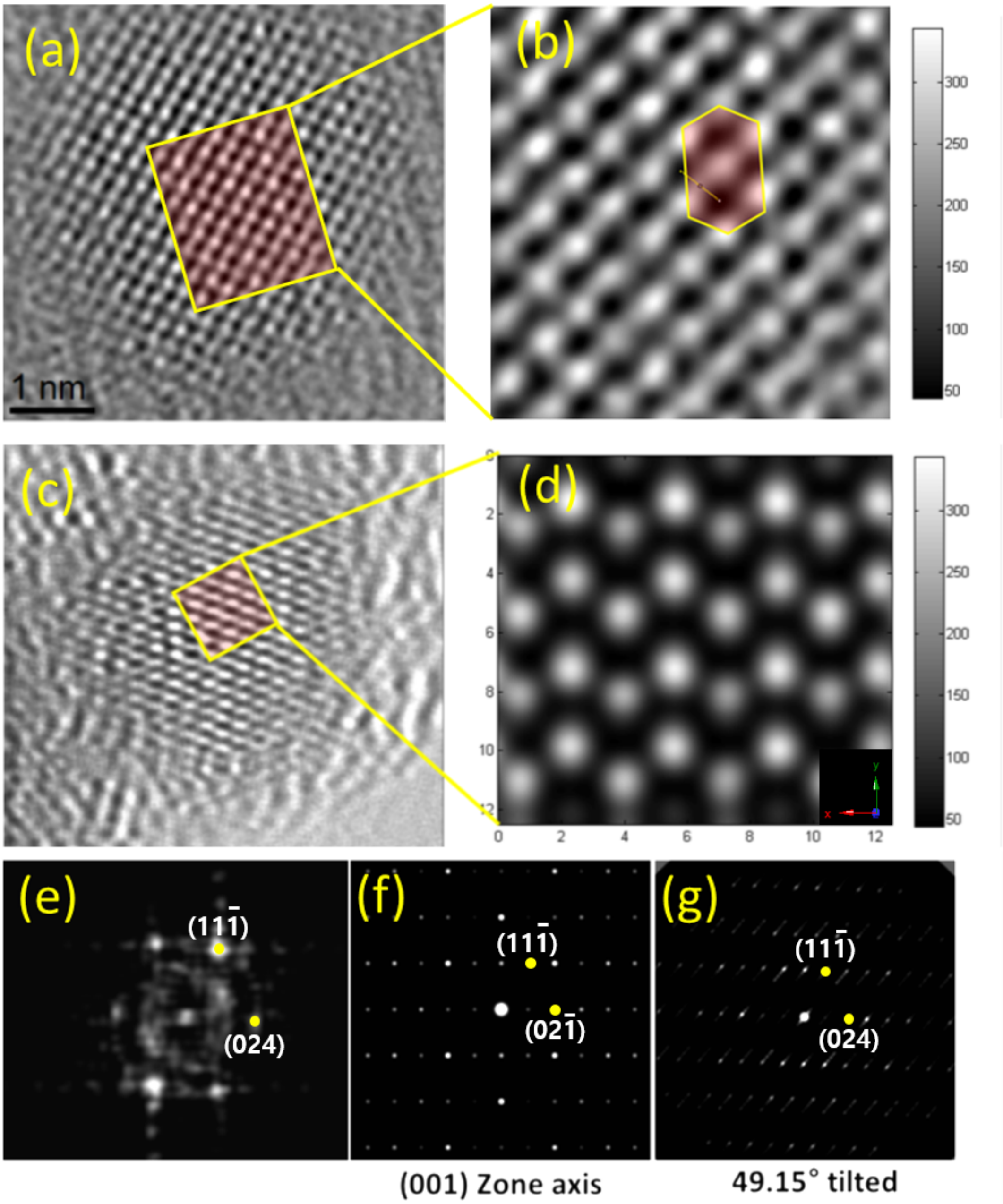

Supplementary Figure 5: Comparison of experimental HRTEM images with the simulated 10$\bar{1}$0 facet image and the relevant EDPs: (a) HRTEM image of the Ni_2P nanoparticle presented in the upper left inset of Figure 5a of the main article. **(b)** Magnified region of the HRTEM image, showcasing the (10$\bar{1}$0) facet. **(c)** HRTEM image of the Ni_2P nanoparticle presented in the lower right inset of Figure 5a of the main article. The marked area matches with the (10$\bar{1}$0) facet, considering a 49.15° tilt angle of the zone axis in respect to the (001) plane. **(d)** Simulated image of the (10$\bar{1}$0) facet with thickness condition matching 5 nm. **(e)** FFT of the HRTEM image shown in panel (c) of this Figure. **(f)** The EDP of the (10$\bar{1}$0) facet in the (001) zone axis. **(g)** The EDP with the zone axis tilted by 49.15°.

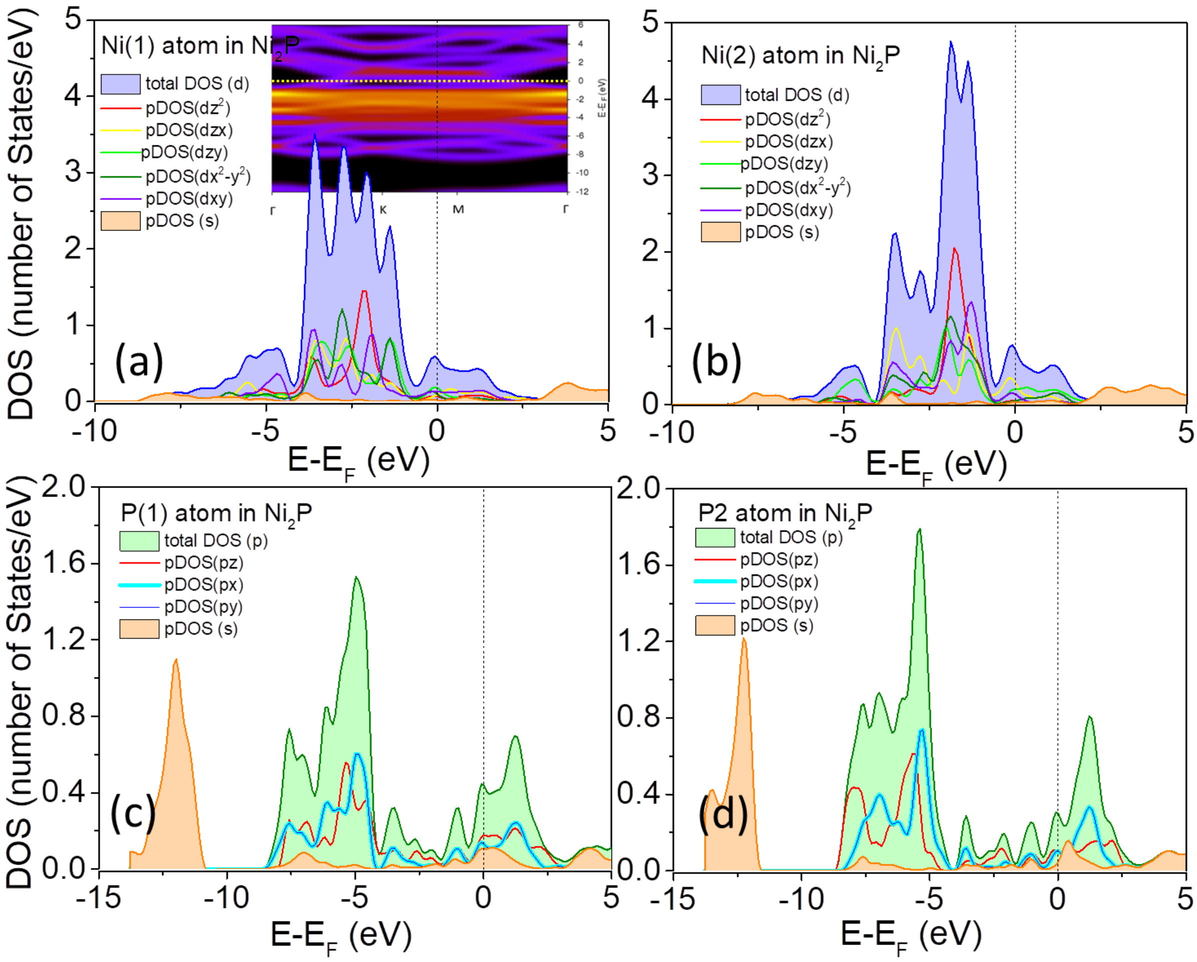

Supplementary Figure 6. **The Density of Energy States (DOS) of bulk Ni_2P. (a, b)** d and s electron pDOS calculated for the two non-equivalent Ni(1) and Ni(2) sites. The inset in the upper left panel shows k-resolved DOS of bulk Ni_2P across the high symmetry points (Γ-K-M-Γ), color-encoded according to the relevant DOS weight; yellow (blue) for high (low) DOS values, respectively. **(c, d)** p and s electron pDOS calculated for the two non-equivalent P(1) and P(2) sites.

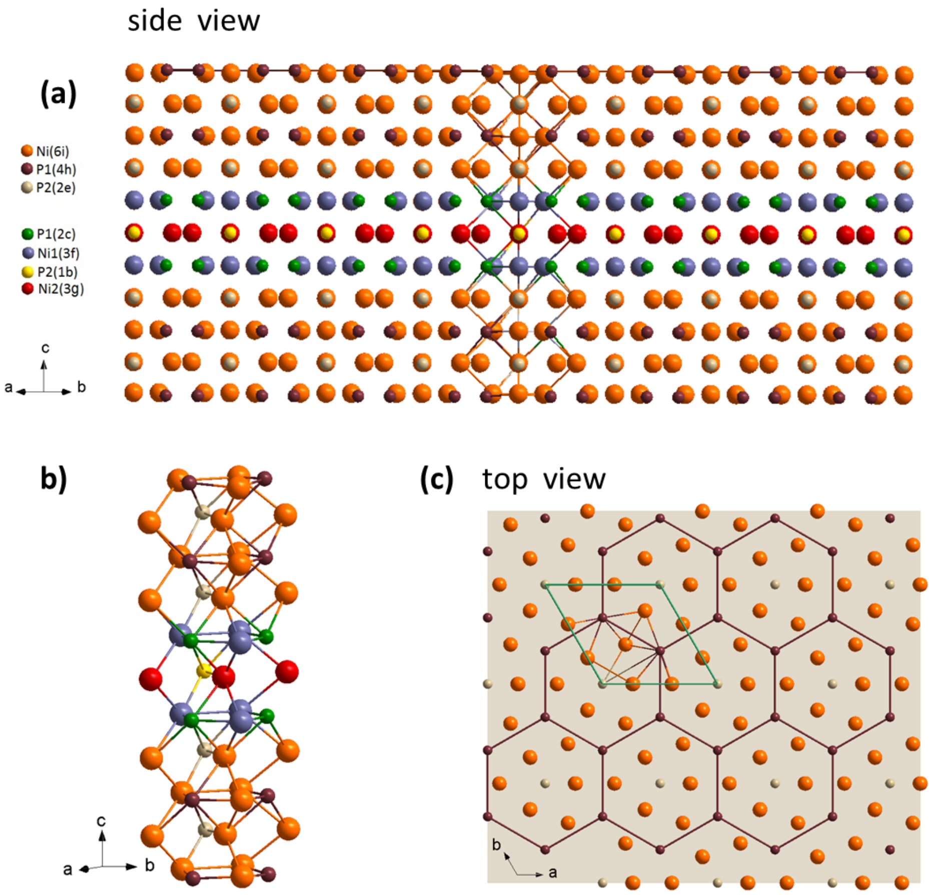

Supplementary Figure 7. Optimized (relaxed) (0001-A) Ni_2P super-cell geometry (1.85 nm slab terminated in the [0001] direction with Ni_2P_3). Optimization was performed on the basis of Broyden–Fletcher–Goldfarb–Shanno (BFGS) algorithm in the Quantum Espresso DFT software [9].

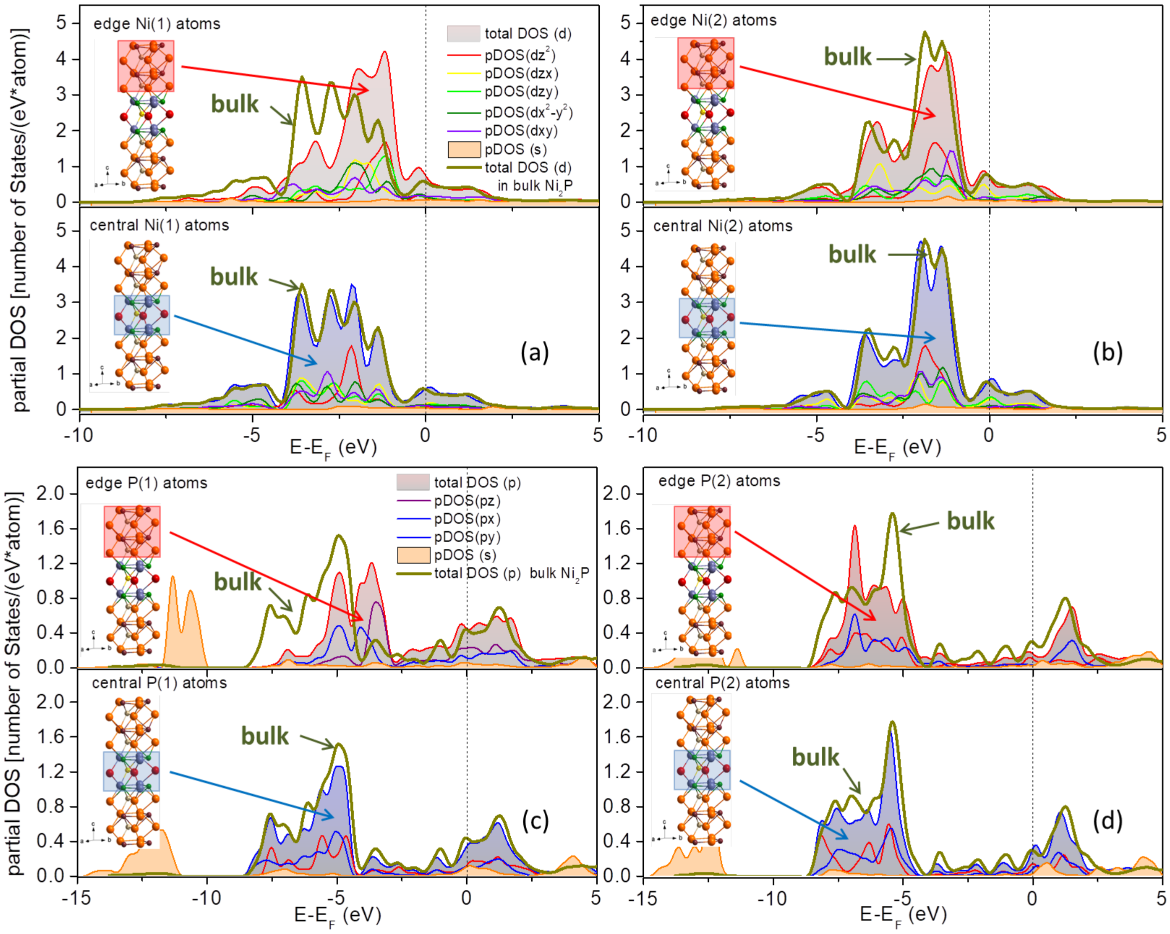

Supplementary Figure 8. The projected electron density of states (pDOS) of the 1.85 nm (0001-A) terminated Ni_2P slab. **(a, b)** Total DOS and d, p, and s electron pDOS of the Ni(1) and Ni(2) sites (i) at the terminating surfaces (red shaded areas) and (ii) in the bulk-like central region of the slab (blue shaded areas). For comparison, the Ni(1) and Ni(2) total DOS of the bulk Ni_2P is shown in olive color. **(c, d)** Total DOS and p, s electron pDOS of the two non-equivalent P(1) and P(2) sites (i) at the terminating surfaces (red shaded areas) and (ii) in the bulk-like central region of the slab (blue shaded areas). The relevant total DOS of the bulk system is shown in olive color.

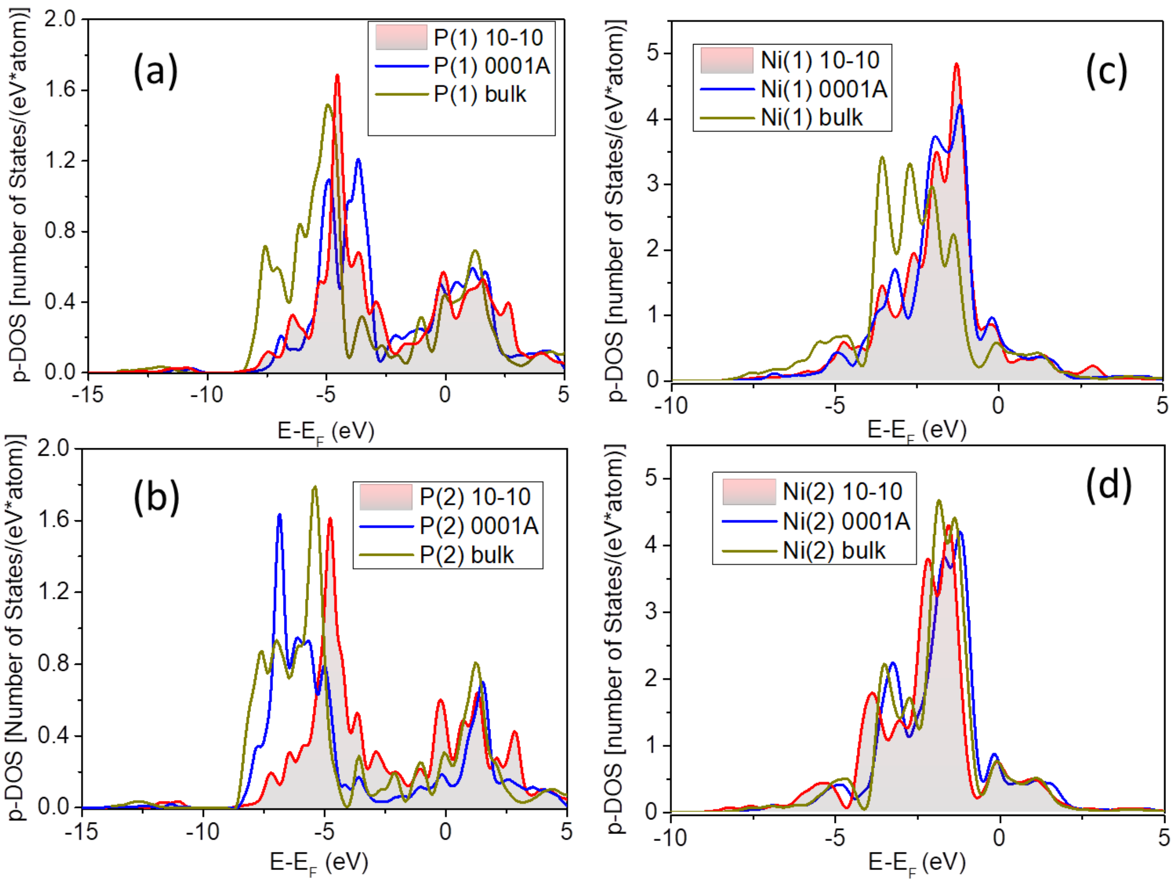

Supplementary Figure 9. The surface electrons pDOS of a ~2.9 nm Ni_2P slab with $(10\bar{1}0)$ termination. **(a, b)** The pDOS of surface p electrons of the two non-equivalent P(1) and P(2) sites in comparison to the (0001-A) terminated slab and the bulk system. **(c,d)** The pDOS of surface d electrons of the two non-equivalent Ni(1) and Ni(2) sites in comparison to the (0001-A)terminated slab and the bulk system.

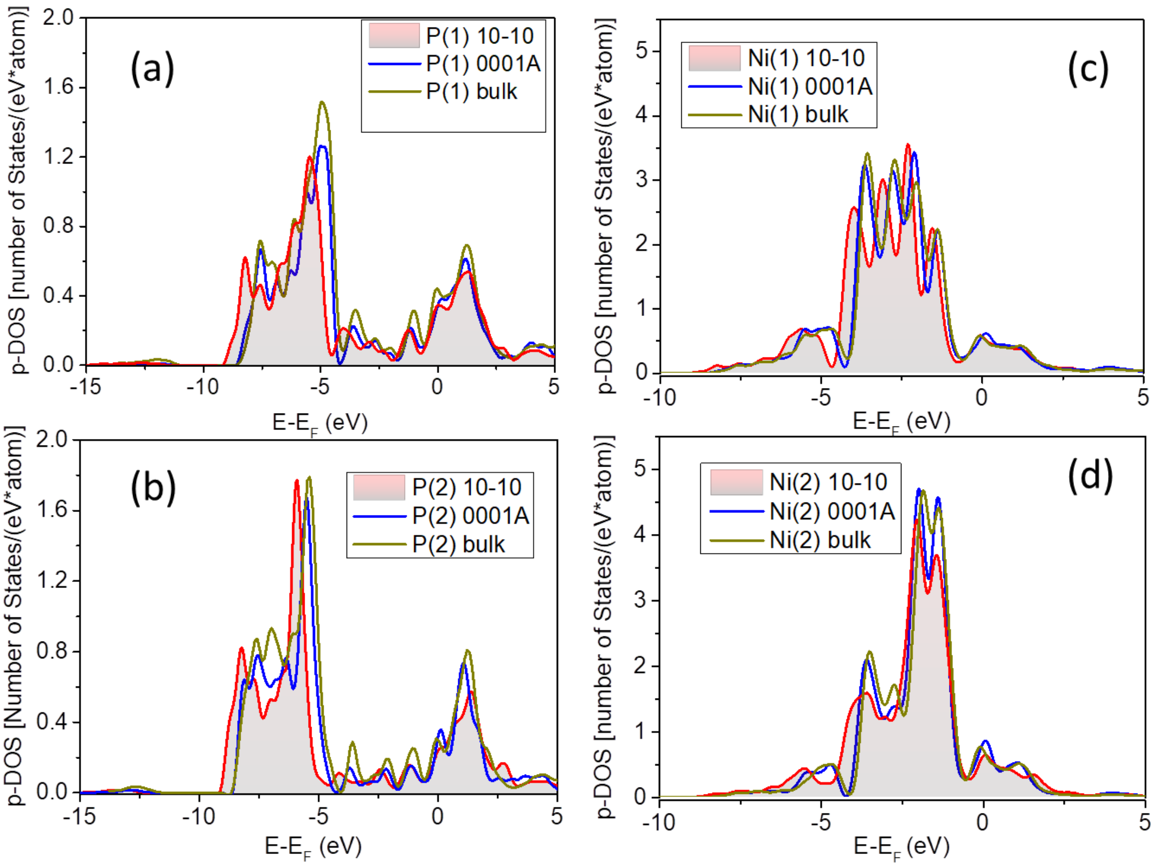

Supplementary Figure 10. The pDOS from the central region of a ~2.9 nm Ni_2P slab with $(10\bar{1}0)$termination. **(a, b)** The pDOS of central bulk-like p electrons of the two non-equivalent P(1) and P(2) sites in comparison to the (0001-A) terminated slab and the bulk system. **(c,d)** The pDOS of central bulk-like d electrons of the two non-equivalent Ni(1) and Ni(2) sites in comparison to the (0001-A)terminated slab and the bulk system.

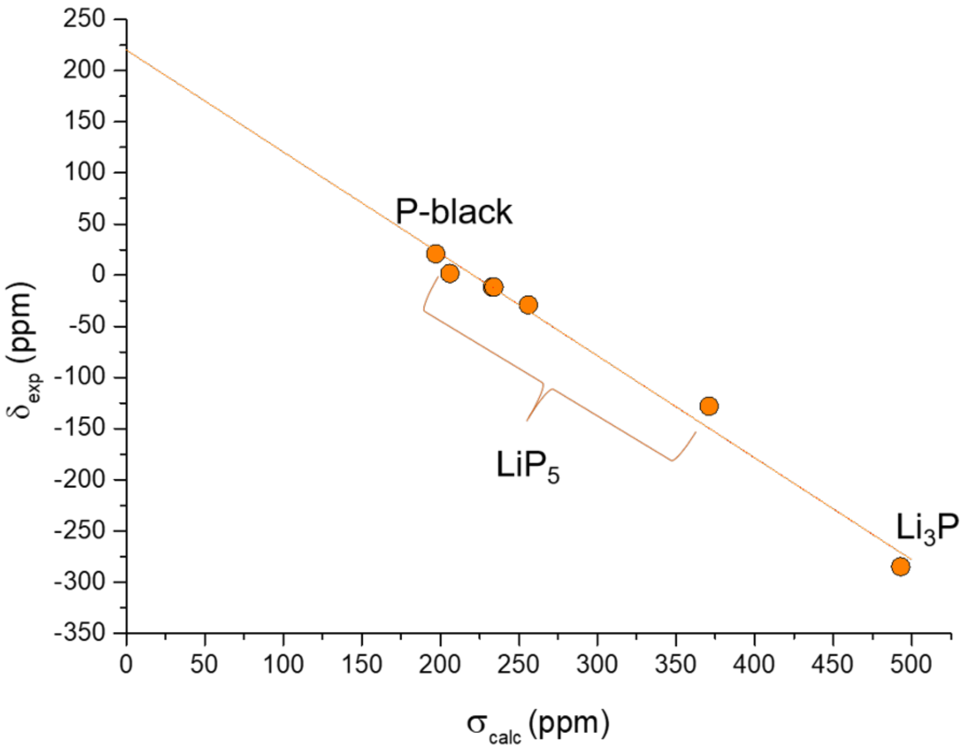

Supplementary Figure 11: Correlation between calculated ^{31}P NMR isotropic shielding and experimental isotropic chemical shift. Data were considered for three representative compounds: Black Phosphorous, LiP_5 and Li_3P. According to the linear regression fit the reference shielding is equal to $\sigma_{ref} = 220.2352$ ppm, and the slope is -0.996.

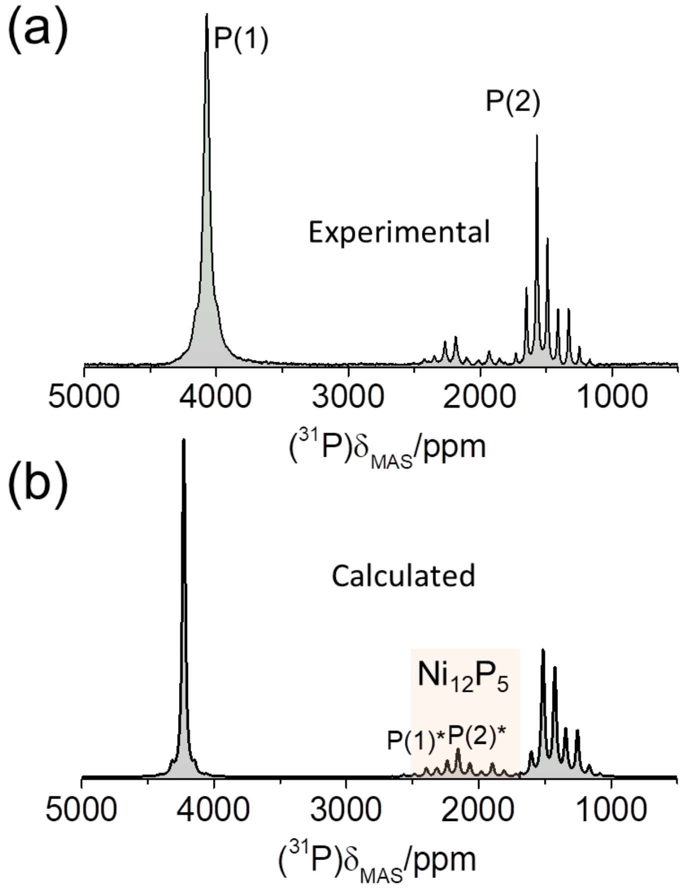

Supplementary Figure 12. Comparison of the experimental and calculated ^{31}P MAS NMR spectra, acquired at spinning rate 14 kHz, by increasing the calculated anisotropies to the experimental values in Supplementary Table 1. All other calculated parameters were kept the same as in Supplementary Table 1. Similar process was applied to the $Ni_{12}P_5$ minority phase. The nice match between calculated and experimental spectra is indication that the orbital anisotropy is underestimated by the DFT calculation.

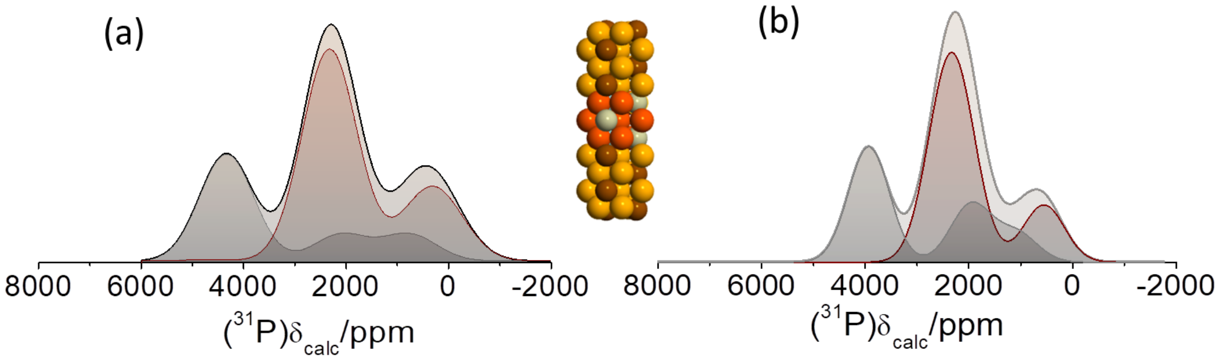

Supplementary Figure 13: Calculated ^{31}P NMR spectrum of the (0001-A) terminated 1.85 nm slab: (a) relaxed with Quantum Espresso (b) relaxed with CRYSTAL. In brown color is shown the contribution from surface phosphorous atoms and grey color the contribution from the central region. Spectra exhibit the same features, while a slight deviation in the P(2) central contribution is apparently coming from the small surface wave functions penetration.

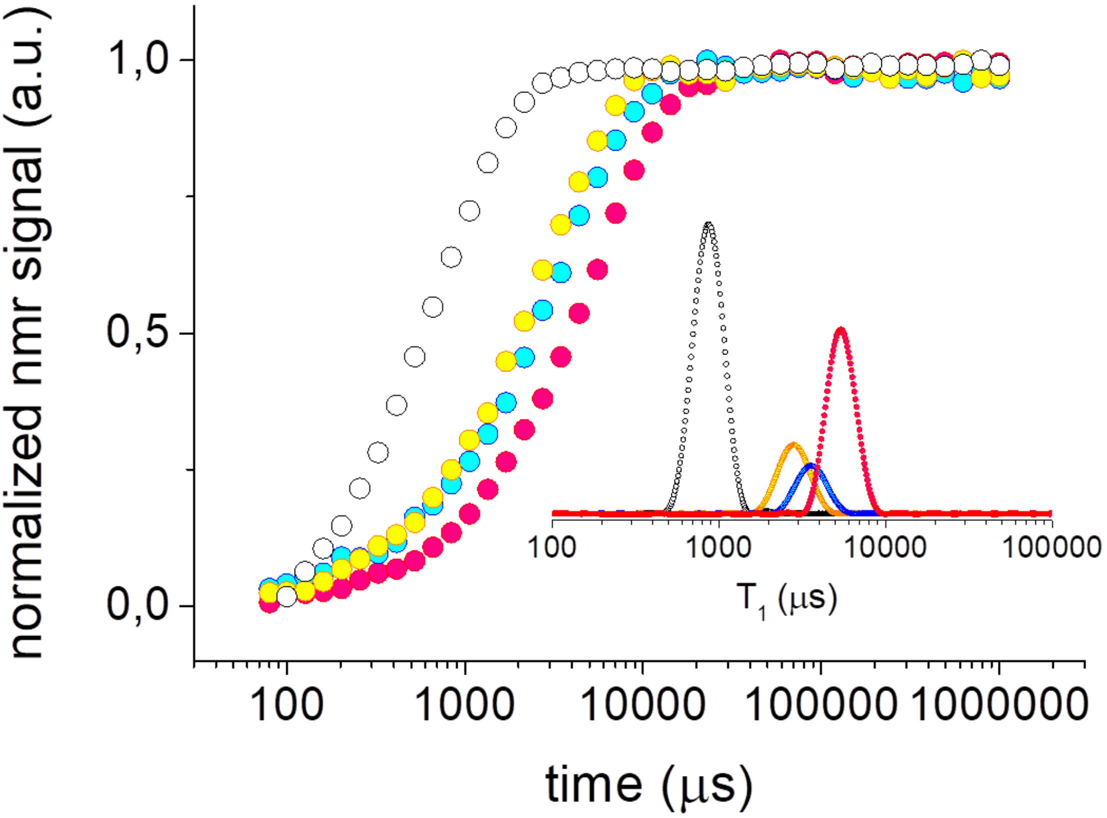

Supplementary Figure 14: Normalized ^{31}P NMR saturation recovery curves of bulk Ni_2P, obtained with a π/2-τ-π/2 pulse sequence, at frequencies: 4058 ppm, 2232 ppm, 1953 ppm, and 1586 ppm (ordered from short to long relaxation times). The inset shows the corresponding inversions ($g(T_1)$ distributions) at their real signal intensities.

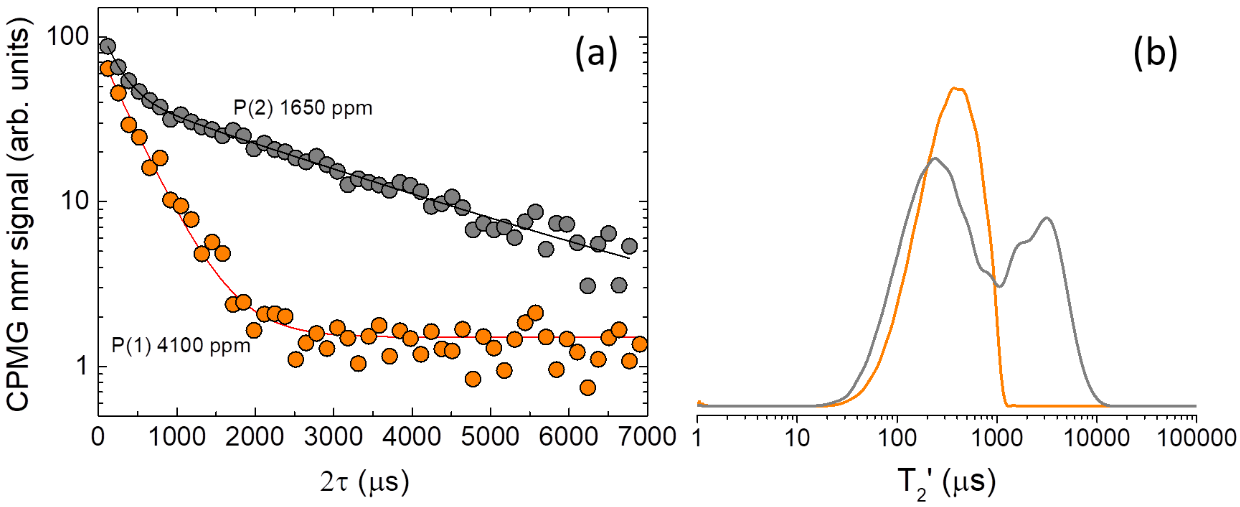

Supplementary Figure 15: (a) CPMG (Carr-Purcell-Meiboom-Gill) ^{31}P NMR spin echo decays of the 12 nm Ni_2P nanocrystalline sample, obtained with a $\pi/2$-(τ-π-τ)$_n$ pulse sequence, at frequencies: 1650 ppm (grey color) and 4100 ppm (orange color). (b) The corresponding inversion distributions (g(T_2').

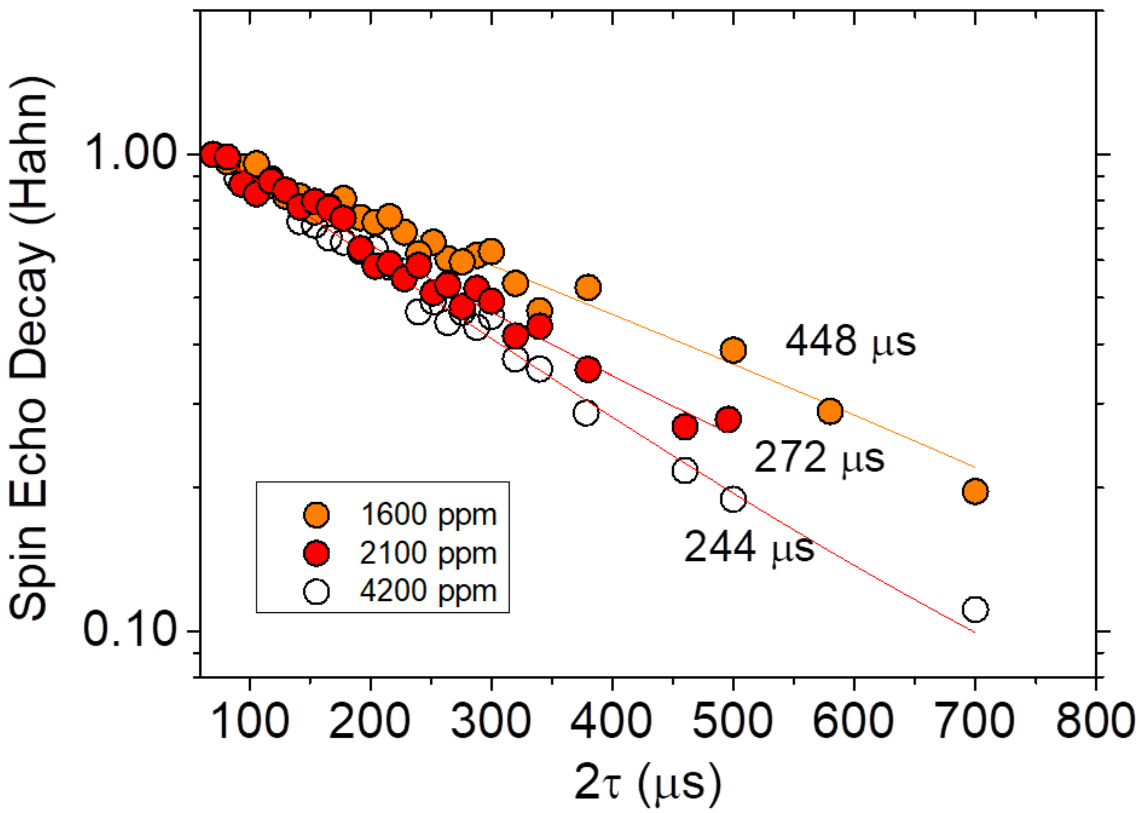

Supplementary Figure 16: ^{31}P NMR Hahn spin echo decays of the 4.3 nm Ni_2P nanocrystalline sample, obtained with a π/2-τ-π pulse sequence, at frequencies: 1600 ppm (orange color) 2100 ppm (red color) and 4200 ppm (no color). The relevant T_2 values in μs are given in the plot.

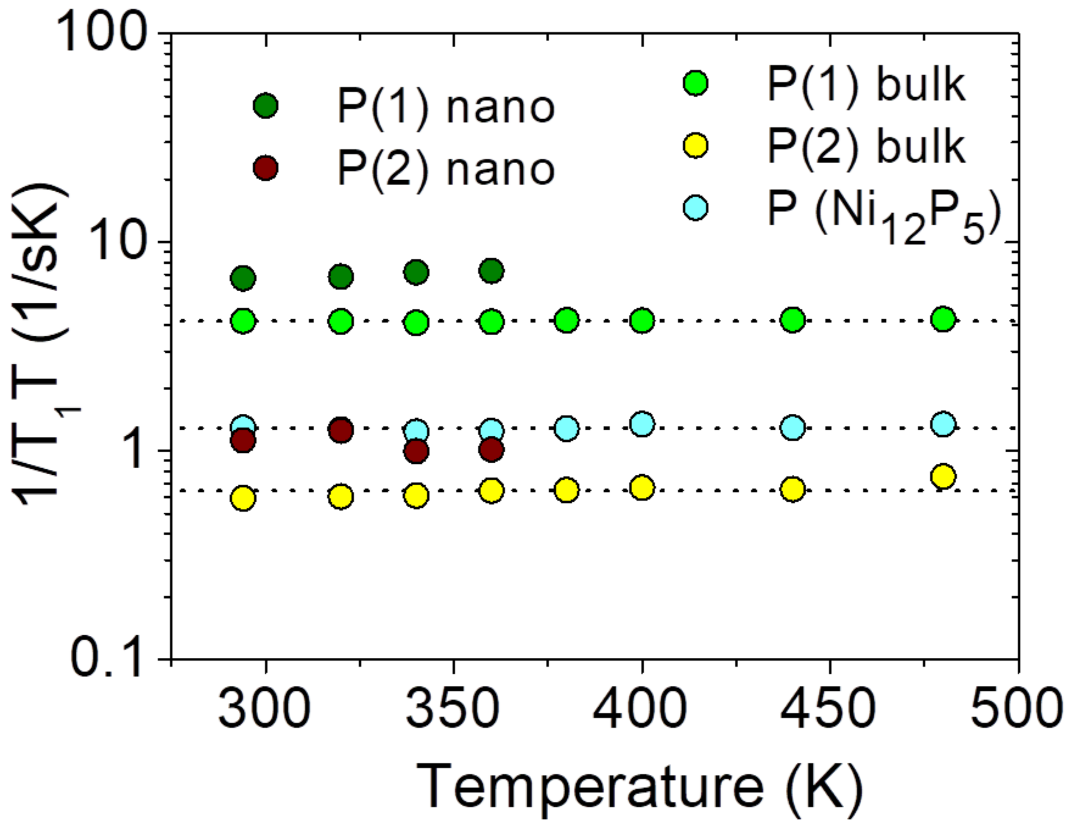

Supplementary Figure 17: Comparison of $1/T_1T$ vs. temperature between bulk Ni_2P and Ni_2P nanoparticles. All plots follow excellently the Koringa relation presented in Supplementary Note 5. The nanoparticles acquire higher $1/T_1T$ values dictating the prevalent role of surface electrons at the nanoscale.

Site	Calculated NMR shift			Experimental NMR shift		
	K_{iso} (calc)/ppm	$\Delta\delta$(calc) / ppm	η	K_{iso} (exp)/ppm	$\Delta\delta$(exp) / ppm	η
**P(1)**	4234	-39.35	0.425	4072.48	-123.7	0.863
**P(2)**	1430	-242.41	0.271	1490	-394.2	0.2

Site	Calculated NMR shielding				
	σ_{orb} (calc)/ppm	σ_{FC} (calc)/ppm	Orbital - σ_{xx}	Orbital - σ_{yy}	Orbital - σ_{zz}
**P(1)**	62.45	-4076	43.76	54.90	88.68
**P(2)**	-146.78	-1063	-249.47	-205.69	14.83

Supplementary Table 1: Comparison of calculated and experimental ^{31}P NMR parameters of the Ni_2P microcrystalline (bulk) sample. The calculated values were referenced according to formula $K_{calc}=\sigma_{ref} - \sigma_{calc}$ with σ_{ref} =220.23 ppm, and $\sigma_{calc} = \sigma_{FC} + \sigma_{dip} + \sigma_{orb}$. Calculations have shown that dipolar contribution is negligibly small. Therefore, only the principal values of the orbital shielding tensor σ_{xx}, σ_{yy}, and σ_{zz} are presented as it is the only source of anisotropy. Shift anisotropies are derived from the Haeberlen [1] convention where $\Delta\delta = \delta_{zz} - (\delta_{xx}+\delta_{yy})/2$ The observed deviation between calculated and experimental shift anisotropies is probably due to sensitivity of the orbital term to the electronic structure of the local environments which can get distorted during relaxation of the unit cell and atomic positions.

Supplementary note 1: Sample characterization

Supplementary Figure 1 presents the XRD analysis of bulk Ni_2P together with the XRD patterns of the three nanoparticle systems presented in the main article. In case of the bulk material, Rietveld refinement [2] was performed with the help of the Fullprof software package [3]. According to the analysis shown in Supplementary Figure 1a, the bulk sample consists of 92.54wt% Ni_2P (P-62m, #189, with cell dimensions equal to a = b = 5.858(2) Å and c = 3.384(5) Å) and 7.46wt% the tetragonal $Ni_{12}P_5$.

In the case of the three nanoparticle systems, the peaks at 2θ of 40.6° (111), 44.5° (201), 47.1° (210), 54.2° (002/300) and 54.8° (211) correspond to the typical Ni_2P characteristic peaks (PDF#03-0953). The crystallite size was measured with (i) TEM statistics and (ii) by measuring the full width at half–maximum (FWHM) of the (111) reflection and applying the Scherrer equation [1] $d = k\lambda(B\cos\theta)^{-1}$, where d is the mean crystallite size in nm, k the Scherrer constant (0.89 for spherical nanoparticles), λ the X–ray wavelength (0.15418 nm), and B the peak width at half–maximum.

Sample	Diameter (nm) (TEM)	Diameter (nm) (Scherrer Equation)
**Commercial Ni_2P**	between 1000 and 100000	
**40 nm Ni_2P**	39.10 ± 13.43	28.66
**12 nm Ni_2P**	11.95 ± 0.91	12.85
**8 nm Ni_2P on RGO**	6.50± 0.50	7.78
**5 nm Ni_2P**	4.32 ± 0.60	4.51

Theoretical XRD patterns of bulk Ni_2P and $Ni_{12}P_5$ were acquired from the relevant cif files, (https://materialsproject.org/materials/mp-21167/, https://materialsproject.org/materials/mp-2790/) with the help of the VESTA software. The acquired XRD patterns were Gaussian broadened to match with the experimental results for the nanoparticles.

Supplementary Figure 2 shows representative TEM images of three extra synthesized Ni_2P nanoparticle systems with a narrow distribution of sizes, ranging between 4 nm – 5 nm,

together with their XRDs. In addition the 40 nm Ni_2P system is shown in Supplementary Figure 2d.

Supplementary Figure 3 displays structural information on the $Ni_{12}P_5$ phase, which very often is co-synthesized as minority phase with Ni_2P. TEM image of the synthesized $Ni_{12}P_5$ nanoparticles is shown in Supplementary Figure 3a. The sample stoichiometry and crystal structure was confirmed by performing Rietveld analysis on the relevant XRD spectrum (Supplementary Figure 3b). According to the crystallographic analysis the $Ni_{12}P_5$ nanoparticles crystallize in the tetragonal phase (I4/m) with lattice constants equal to a=b=8.6447 Å, and c=5.0702 Å [4].

Unequivocally, no $Ni_{12}P_5$ minority phase is present in the synthesized Ni_2P nanoparticles as proved by the XRD comparison in Supplementary Figure 1b.

Finally, Supplementary Figure 4 shows the High Resolution TEM image and the FT-IR of the 12 nm nanocrystalline sample. The measured distance between the lattice fringes in the TEM image corresponds to 2.21Å, which matches with the 111 plane in Ni_2P. No information about the eventual facets can be revealed from the TEM and the relevant XRD presented in Supplementary Figure 1b. The FT-IR spectrum of the Ni_2P shows no obvious peaks around 3000 cm^{-1}, which indicate that the ligand on the surface of the nanoparticles was effectively removed (peaks at ~3000 cm^{-1} are assigned to the C-H stretching modes of the CH_2 and CH_3 groups, in oleylamine (trioctylphosphine as well). The FT-IR spectra of trioctylphosphine and oleylamine were taken from:

https://www.chemicalbook.com/SpectrumEN_4731-53-7_IR1.htm

https://www.chemicalbook.com/Spectrum_112-90-3_IR1.htm.

Supplementary note 2: DFT calculations of relaxed structures and electron DOS

DFT calculations of the relaxed structures and electron DOS were performed using the QUANTUM ESPRESSO Simulation Package code (QE 5.4.0) [5]. The Perdew-Burke-Ernzerhof exchange-correlation functional with the generalized gradient approximation [6], [7] was used. The Projector-Augmented Wave approach (PAW) [8] has been used together with plane wave basis sets. In case of bulk Ni_2P, calculations were performed by inserting in the DFT calculations the lattice parameters from the Rietveld analysis. The kinetic energy cutoff of 480 eV was used and the Brillouin zone was sampled by a 12x12x12 Monkhorst-Pack mesh [9]. The z-axis was taken as normal to the surface.

The Ni_2P (0001-A) facet was modeled with a Ni_2P super-cell comprised of 5 Ni_2P unit cells

(successive Ni_3P_2 and Ni_3P layers), terminated with Ni_3P_2 layer (super-cell with 50 atoms). Calculations were performed allowing structural relaxation on the basis of Broyden–Fletcher–Goldfarb–Shanno (BFGS) algorithm [10]. The kinetic energy cut-off was set 480 eV and a 12x12x1 Monkhorst-Pack mesh [9] was used. The optimized super-cell geometry is presented in Supplementary Figure 7. Calculations have shown that the structure at the center of the supercell is equivalent with that of bulk Ni_2P, while at both edges the local crystal symmetry changes and only one type of Ni atom is present close to the Ni(2) symmetry. At the same time the relevant Phosphorous atoms stretch out of the terminal plane as defined by the Nickel atoms, with distance $\mathbf{\Delta z} \approx \mathbf{0.15}$Å, while at center $\mathbf{\Delta z} \approx \mathbf{0}$.
Similarly, the (0001-B)facet (Ni_2P slab with Ni_3P termination – super-cell with 49 atoms), the (0001-A-P) (0001-A) slab with P adatom on top of the Ni_3P_2 terminating layer – super-cell with 52 atoms), as well as the $(10\bar{1}0)$ facet (super-cell with 54 atoms), were all structurally relaxed in the way described above. For comparison, the (0001-A) terminated slab was optimized with the CRYSTAL17[11] package using Anisotropic Monkhorst-Pack reciprocal space meshes[9] with sets of shrinking factors (2,2,1) within the PBE level of theory[6]. The Knight shifts of the optimized structures were then calculated with the Wien2k package as described in the main article.
Supplementary Figures 6, 8, 9, and 10 show the projected electron DOS of the Ni and P electron states in bulk Ni_2P, (0001-A), and $(10\bar{1}0)$ terminated slabs.
In both (0001-A), and $(10\bar{1}0)$ terminated slabs, the pDOS of the surface Ni(1) d-electron orbitals shifts closer to the Fermi level E_F, becoming similar to the relevant pDOS of the Ni(2) atoms. This is induced by the formation of a unique Ni crystal environment at the terminating surfaces, as shown in Supplementary Figure 7. Notably, according to the d-band theory [12], the shift of the surface d-electron bands towards the Fermi level indicates enhancement of the catalytic activity.
At the central regions of the slabs, the d-electron and p electron DOS is similar to the relevant DOS of bulk Ni_2P (Supplementary Figure 10). This legitimates to simulate calculated NMR spectra of nanoparticles with the sum of the NMR spectra from different facets and inner bulk-like states.

Supplementary Note 3: Correlation between experimental isotropic ^{31}P NMR chemical shift and the DFT-calculated isotropic magnetic shielding.

In order to obtain the reference isotropic ^{31}P magnetic shielding σ_{ref}, NMR calculations were

performed on three materials with experimental chemical shift values δ_{exp} taken from ref. [13]. Crystallographic data for these materials were acquired from https://materialsproject.org to perform the NMR DFT calculations with Wien2k. The data in Supplementary Figure 11 are plots of the calculated isotropic magnetic shielding as function of the experimental isotropic chemical shifts; The orange line is the fit to the plot according to formula $\delta_{exp} = 220.223 - 0.996\sigma_{calc}$. The calculated reference shielding σ_{ref}, defined as the intercept of the best-fit line with the vertical axis, was found to be $\sigma_{ref} = 220.23$ ppm.

Supplementary Note 4: Details on the inversion to acquire the ^{31}P NMR

The ^{31}P NMR spin-lattice relaxation time (T_1) distribution function $g(T_1)$, was acquired by modeling the experimental saturation recovery curves (main panel in in Supplementary Figure 14) with a Fredholm integral equation of the first kind [14,15], $\frac{M(t)}{M(0)} = \int_0^{+\infty} k_0(T_1, t) g(T_1) d(log_{10} T_1)$ (S1).

Here, $\frac{M(t)}{M(0)}$ is the normalized signal intensity and the Kernel function $k_0(T_1, t)$ is equal to $k_0(T_1, t) = 1 - exp\left(-\frac{t}{T_1}\right)$. This equation is transformed into a discrete vector matrix formulation $M = K_0 g$, which can be inverted by implementing a non-negative Tikhonov regularization algorithm to acquire $g(T_1)$, as nicely shown in the inset of Supplementary Figure 14 .

The contour plots in Figure 1c of the main article is made out of consecutive $g(T_1)$ curves, at different resonance frequencies, covering the whole ^{31}P NMR spectra. Soft pulses were used so that each time a narrow frequency bandwidth was irradiated.

The ^{31}P NMR T_2' relaxation time distribution function $g(T_2')$, was acquired by modeling the experimental CPMG (Carr-Purcell-Meiboom-Gill) spin-echo decay curves (see example in main panel of Supplementary Figure 15) in a similar way as for $g(T_1)$, by using the Kernel function $k_0(T_2', t) = exp\left(-\frac{t}{T_2'}\right)$. The contour plots in Figure 7a of the main article is made out of consecutive $g(T_2')$ curves, at different resonance frequencies, covering the whole ^{31}P NMR spectrum of the 12 nm nanocrystalline sample.

Supplementary Note 5: ^{31}P NMR spin-lattice relaxation T_1 of Ni_2P bulk material and nanoparticles follows Korringa relation

Supplementary Figure 17 compares $\left(\frac{1}{T_1T}\right)$ vs. temperature between microcrystalline (bulk) Ni_2P and Ni_2P nanoparticles. In both systems $\left(\frac{1}{T_1T}\right)$ is shown to be constant by varying temperature, following the so called Korringa relation.

In conventional metals, the relaxation of nuclear spins occurs mainly via the spin fluctuations of the conduction electrons, predominately through the Fermi–contact interaction of the s-electrons [16], while orbital and dipolar contributions are small. In such case, and for non-interacting electrons, the temperature dependence of T_1 is given by the well-known Korringa relation [17]:

$$\left(\frac{1}{T_1T}\right)_s = \frac{4\pi k_B}{\hbar}(\gamma_e\gamma_n\hbar^2)^2\left[\frac{8\pi}{3}\left\langle|\varphi_s(0)|^2\right\rangle_F \mu_B\right]^2 \left[N(E_F)\right]^2 \qquad \text{(S2)} \quad ,$$

where k_B is the Boltzmann constant, T is the temperature, and γ_e and γ_n are the gyromagnetic ratios of the electron and nuclear spins, respectively. The Knight shift K (Equation (1) in the main article and the relaxation time T_1 for a system of non-interacting conduction electrons coupled to the nucleus with the Fermi contact interaction can be combined to give the *Korringa product* relation[15]

$$\left(\frac{1}{T_1TK^2}\right)_s = \frac{4\pi k_B}{\hbar}\left(\frac{\gamma_n}{\gamma_e}\right)^2 \qquad \text{(S3)}$$

The above relation shows that when $\left(\frac{1}{T_1T}\right)$ is divided by K^2 for simple metals the resulting product is made up of physical constants independent of the other properties of the metal.

Korringa relation is considered as direct proof of the metallic nature of the system.

The experimental $\left(\frac{1}{T_1T}\right)$ Koringa relaxation rate for the Ni_2P nanoparticles was found to obey the Korringa relation as indicated by the constant value of $\left(\frac{1}{T_1T}\right)$ in Supplementary Figure 17. This is evidence that - as in the bulk case – the relaxation of surface nuclei is governed by conduction electrons. It is further observed that the value of $\left(\frac{1}{T_1T}\right)$ for the nanoparticles is enhanced with respect to the corresponding one of the bulk metal. This is due to the fact that

the T_1 relaxation of the nanoparticles is modulated by the electronic fluctuations of the surface spins generally regarded as non-collinear or disordered. Also the presence of dangling bond at surfaces may substantially enhance the $\left(\frac{1}{T_1}\right)$relaxation rate.

Supplementary References

1. U. Haeberlen, *Advances in Magnetic Resonance*; Suppl. 1; J. S. Waugh, Ed.; Academic Press: New York (1976).
2. Rietveld, H. M. Photoemission Studies of Ordered Pd Overlayers on Au(111). Implications for CO Chemisorption. *J. Appl. Cryst.* **2**, 65–71 (1969).
3. https://www.ill.eu/sites/fullprof/.
4. Rundqvist, S. and Larsson, E., The Crystal Structure of $Ni_{12}P_5$, *Acta Chem. Scand.,* **13**, 551-560 (1959).
5. Ceresoli, D.; Chiarotti, G. L.; Cococcioni, M.; Dabo, I.; Dal Corso, A.; Fabris, S.; Fratesi, G.; de Gironcoli, S.; Gebauer, R.; Gerstmann, U.; Gougoussis, C.; Kokalj, A.; Lazzeri, M.; Martin-Samos, L.; Marzari, N.; Mauri, F.; Mazzarello, R.; Paolini, S.; Pasquarello, A.; Paulatto, L.; Sbraccia, C.; Scandolo, S.; Sclauzero, G.; Seitsonen, A. P.; Smogunov, A.; Umari, P.; Wentzcovitch, R. M. Quantum Espresso: a modular and open-source software project for quantum simulation of materials. *J. Phys.: Cond. Matt.* **21**, 395502 (2009).
6. Perdew, J. P.; Burke, K.; Ernzerhof, M. Generalized Gradient Approximation Made Simple. *Phys. Rev. Lett.* **77**, 3865 (1996).
7. Perdew, J. P.; Burke, K.; Ernzerhof, M. Generalized Gradient Approximation Made Simple. *Phys. Rev. Lett.* **78**, 1396 (1997).
8. Blöchl P. E. Projector augmented-wave method. *Phys. Rev. B* **50**, 17953 (1994).
9. Monkhorst H. J.; Pack J. D. Special Points for Brillouin-Zone Integrations. *Phys. Rev. B* **13**, 5188 (1976).
10. Dennis J. E.; Schnabel R. B. Numerical Methods for Unconstrained Optimization and Nonlinear Equations. Prentice-Hall, Englewood Cliffs, NJ, (1983).
11. Dovesi, R. et al. Quantum-mechanical condensed matter simulations with CRYSTAL. *WIREs Comput. Mol. Sci.* **8**, e1360 (2018).
12. Hammer B.; Norskov J. K. Theoretical Surface Science and Catalysis—Calculations and Concepts. *Advances in Catalysis* **45**, 71-129 (2000).

13. Mayo, M., Griffith, K. J., Pickard, C. J. & Morris, A. J. Ab initio study of phosphorus anodes for lithium- and sodium-ion batteries. *Chem. Mater*. **28**, 2011–2021 (2016).
14. Mitchell, J., Chandrasekera, T. C. & Gladden, L. F. Numerical estimation of relaxation and diffusion distributions in two dimensions. Prog. Nucl. Magn. Reson. Spectroscopy 2012, **62,** 34-50.
15. Day, I. J. On the inversion of diffusion NMR data: Tikhonov regularization and optimal choice of the regularization parameter. *Journal of Magnetic Resonance* **211,** 178-185 (2011).
16. Slichter, C. P. Principles of Magnetic Resonance. *Springer, Berlin*, (1990).
17. Korringa, J. Nuclear magnetic relaxation and resonance line shift in metals. *Physica* **16**, 601–610 (1950).
